# At the border of the unknown—a plea for curiosity

**DOI:** 10.1007/s00709-019-01478-9

**Published:** 2020-01-10

**Authors:** Peter Nick

**Affiliations:** Botanical Institute, Karlsruher Institut für Technologie, Karlsruhe, Germany

Students of biology usually learn from models depicted as neat illustrations in textbooks. These models are so common that they are easily perceived as reality. However, reality is never evident, but has to be inferred by searching. “Really, not from the beginning the Gods have revealed everything to the mortals, but gradually they have to find it, searching, what is better”—these words by the Greek philosopher Xenophanes (Gentili and Prato 1988) were written more than 2500 years ago, but still appropriately describe, what science is about. Science has, thus, much in common with bacterial chemotaxis—it is a heuristic strategy to find out something about reality. The bacteria perform quite efficiently in this respect, basically using a very simple scouting strategy: swim straight as long as the signal is increasing; change direction by tumbling, when this is not any longer the case (Adler 1966). Translated into the world of science: go ahead with a model, as long as your experimental data are consistent with the implications drawn from the model; overthrow the model, when you find out that empiry and prediction fall apart. So, “tumbling”, searching for new directions, is essential for the progress of science. However, our current system of funding is not always encouraging the search for new directions, but exerts a certain conformistic pressure upon going on with the established lines of research. Three contributions to the current issue show exemplarily that it is rewarding to go own paths, even if they may appear exotic at first sight.

The interest in trees has recently been boosted by the understanding that we will not be able to constrain climate change if we fail to improve the performance of forests. From an experimental point of view, trees are not easy to approach, since their long life cycle collides with the conditions of the usually short-lived grants that shape biological research in most countries. To work with Arabidopsis is definitely easier than to work with spruce. To observe tree physiology, both patience and a long breath are needed, and this may be one reason why most of the literature dates back to the 1970s or 1980s of the last century. In their contribution to the current issue, Ovsyannikov and Koteyeva (2020) revisit a curious phenomenon that has been described long time ago in conifers (Soikkeli 1980): chloroplasts in the mesophyll change their position depending on the season, which is interpreted as photoprotective response to avoid oxidative damage during winter time. To get insight into such a phenomenon is far from easy, since one cannot study this under laboratory conditions. The authors have therefore used an experimental design, where they let Nature conduct the experiments: they studied mesophyll structure over three vegetational periods in two geographic locations that differ in the temporal progression of temperature drop, and in two *Picea* species to extract correlative data that allow to infer the mechanisms. Using tomography, they reconstruct the architecture of mesophyll cells and describe peculiar cytoplasmic strands connecting the two lateral walls, where organelles such as nucleus and chloroplasts move to and fro, depending on the season. They not only come up with very clear reconstructions of this phenomenon, but also are able to quantify this over their experimental variants, showing that it is the rate of temperature drop that serves as input for the reorganization. This work is of high relevance for society, because climate change renders seasonal transitions progressively erratic, such that cold acclimation in temperate and boreal regions is at stake. This work allows to define cellular parameters for cold acclimation of trees, which is the base of any adaptive measures in forestry.

Also, the contribution by Decou et al. (2020) to the current issue deals with trees, this time poplar, addressing the phenomenon of so-called tension wood. This wood type is able to compensate tensile forces, which is not only relevant to adjust the stem against the challenges of wind and imbalanced forces, but also to maintain vertical growth. Tension wood shows specific cellular features, such as a gelatinous layer in the secondary walls of xylem fibres. To study the genesis and molecular events behind this important phenomenon requires an experimental system that allows to induce tension wood in a controlled manner, which in turn is only possible, if one can rely upon standardized material. This is difficult to achieve with trees grown under natural conditions. The authors have therefore established an in vitro poplar system, where they can induce tension wood at an early stage of development (3 months). Using histochemistry, they can demonstrate the formation of tension wood. In the next step, they dissect the cell wall components based on immunocytochemistry and find different polymers including crystalline cellulose, and also arabinogalactan proteins and rhamnogalacturonans. Similar studies on tension wood have demonstrated the absence of xylans and xyloglucans in tension wood (Bowling and Vaughn 2008). This can also be confirmed for the poplar in vitro system, supporting that even molecular features of tension wood can be reproduced by this experimental system. A surprising observation is the observation of extensins, cell-wall proteins that are involved in cell-wall loosening. Thus, this experimental system allows a new, even molecular, approach to a phenomenon that so far appeared difficult to target in laboratory experiments.

The contribution by Cui et al. (2020) in the current issue gives a timely update on a curious phenomenon, so-called exocytotic vesicles. While in the meantime well established in animal cells and recognized as major factor in cell-cell communication, their existence had been even denied for plant cells, because they are surrounded by a cell wall. Meanwhile, it is clear that exocytic vesicles exist in plant cells as well and can arise from unconventional forms of secretion, such as exocyst-positive organelle, multivesicular body, and autophagosome-driven forms of exocytosis (Robinson et al. 2016). The current review is focussed on the role of these exosomes in plant-pathogen interaction and symbiosis, where, for instance, small RNAs are secreted that can enter the fungal invader and suppress there the expressions of genes for virulence factors, establishing a new layer of host-pathogen warfare. This fascinating new facet of organismic interaction can only be understood, when one knows the cellular mechanisms leading to the genesis of extracellular vesicles and the regulation of this process. The authors have been dissecting the ramifications of plant vesicle traffic over years and, therefore, are able to integrate the phenomenological data into a conceptual framework that will help to explore this new field also on the molecular level.

To “tumble” rather than to swim straight requires a considerable degree of courage and audacity. It means to leave the beaten track and follow curiosity, inspired by a new idea, not knowing, where it will lead to, or whether it will lead somewhere at all. The driving force is often the mere desire to find something new. This brings experimental and conceptual challenges and often makes life of the respective researcher far from easy. It should be kept in the general consciousness that these pioneers contribute to the development of science as a whole. Science is searching and we need more “tumbling”, if we want to advance.
